# Bursting with Potential: How Sensorimotor Beta Bursts Develop from Infancy to Adulthood

**DOI:** 10.1523/JNEUROSCI.0886-23.2023

**Published:** 2023-12-06

**Authors:** Holly Rayson, Maciej J. Szul, Perla El-Khoueiry, Ranjan Debnath, Marine Gautier-Martins, Pier F. Ferrari, Nathan Fox, James J. Bonaiuto

**Affiliations:** ^1^Institut des Sciences, Cognitives Marc Jeannerod, Centre National de la Recherche Scientifique Unité Mixte de Recherche 5229, Bron, 69500, France; ^2^Université de Lyon, Université Claude Bernard Lyon 1, Lyon, 69100, France; ^3^Center for Psychiatry and Psychotherapy, Justus-Liebig University, Giessen, 35394, Germany; ^4^Department of Human Development and Quantitative Methodology, University of Maryland, College Park, Maryland, 20742; ^5^Inovarion, Paris, 75005, France

**Keywords:** action observation, beta burst, EEG, infant reaching and grasping, sensorimotor cortex

## Abstract

Beta activity is thought to play a critical role in sensorimotor processes. However, little is known about how activity in this frequency band develops. Here, we investigated the developmental trajectory of sensorimotor beta activity from infancy to adulthood. We recorded EEG from 9-month-old, 12-month-old, and adult humans (male and female) while they observed and executed grasping movements. We analyzed “beta burst” activity using a novel method that combines time-frequency decomposition and principal component analysis. We then examined the changes in burst rate and waveform motifs along the selected principal components. Our results reveal systematic changes in beta activity during action execution across development. We found a decrease in beta burst rate during movement execution in all age groups, with the greatest decrease observed in adults. Additionally, we identified three principal components that defined waveform motifs that systematically changed throughout the trial. We found that bursts with waveform shapes closer to the median waveform were not rate-modulated, whereas those with waveform shapes further from the median were differentially rate-modulated. Interestingly, the decrease in the rate of certain burst motifs occurred earlier during movement and was more lateralized in adults than in infants, suggesting that the rate modulation of specific types of beta bursts becomes increasingly refined with age.

**SIGNIFICANCE STATEMENT** We demonstrate that, like in adults, sensorimotor beta activity in infants during reaching and grasping movements occurs in bursts, not oscillations like thought traditionally. Furthermore, different beta waveform shapes were differentially modulated with age, including more lateralization in adults. Aberrant beta activity characterizes various developmental disorders and motor difficulties linked to early brain injury, so looking at burst waveform shape could provide more sensitivity for early identification and treatment of affected individuals before any behavioral symptoms emerge. More generally, comparison of beta burst activity in typical versus atypical motor development will also be instrumental in teasing apart the mechanistic functional roles of different types of beta bursts.

## Introduction

Modulation of neural activity in different frequency bands is a classic signature of many sensory, motor, and cognitive processes. However, the generative mechanisms that drive this activity and the computational functions that band-specific neural activity subserves remain unclear. Developmental research provides a unique opportunity to elucidate these processes by allowing investigation into the unfolding relationship between frequency-specific neural activity and emerging abilities in various domains (Munakata et al., 2004). Very little is known about the development of neural activity in beta band (Cuevas et al., 2014; Perone and Gartstein, 2019), despite its crucial role in sensorimotor and cognitive control in adults (Pfurtscheller and Neuper, 1997; Järveläinen et al., 2004).

Traditionally, beta activity was thought to occur as a sustained oscillation with slow task-related amplitude modulations. However, at the single-trial level, beta activity predominantly occurs as transient bursts rather than oscillations (Feingold et al., 2015; Sherman et al., 2016; Little et al., 2019), changes in burst probability closely track trial-averaged beta power (Feingold et al., 2015; Little et al., 2019), and burst timing is highly predictive of behavior (Shin et al., 2017; Little et al., 2019; Wessel, 2020; Diesburg et al., 2021; West et al., 2023). This suggests that bursts are more informative about sensorimotor processing than beta power, and that averaging bandpass-filtered power over trials may obscure important features and functions of beta activity.

Bursts can be highly diverse in terms of time-frequency-based features, such as power, peak frequency, and frequency span (Feingold et al., 2015; Zich et al., 2020; Duchet et al., 2021; Enz et al., 2021; Szul et al., 2022), as well as in waveform shape in the temporal domain (Szul et al., 2022). In adult sensorimotor cortex, beta bursts have a stereotyped mean waveform shape (Baker et al., 1997; Sherman et al., 2016; Cole et al., 2017; Cagnan et al., 2019; Karvat et al., 2020; Kosciessa et al., 2020; Bonaiuto et al., 2021; Brady and Bardouille, 2022), but individual burst waveforms are highly diverse and occur within motifs, which are differentially rate-modulated before, during, and after movements (Szul et al., 2022). It has been suggested that different types of beta bursts might therefore play different roles in sensorimotor processes (Szul et al., 2022), but it is not yet known what these processes are.

The first year of life is marked by rapid and significant changes in perceptual and motor abilities (Adolph and Franchak, 2017; von Hofsten and Rosander, 2018), making this an ideal age range to probe the relationship between beta activity and movement. Very few EEG studies have looked at sensorimotor beta power in early development (Samson-Dollfus et al., 1983; Ogawa et al., 1984; van Elk et al., 2008; Meyer et al., 2011, 2016; Niemarkt et al., 2011), although studies with young children have demonstrated movement-related decreases in beta power (Gaetz et al., 2010; Cheyne et al., 2014; Liao et al., 2015; Bryant and Cuevas, 2019). We recently demonstrated that beta activity does indeed appear “bursty” in 12-month-olds (Rayson et al., 2022); but if different types of beta bursts are reflective of different sensorimotor processes, they may be differentially rate-modulated as these processes develop in infancy.

Here, we analyzed previously collected EEG recordings obtained during execution and observation of a reaching-and-grasping action in 9- and 12-month-old infants, and an adult comparison group (Cannon et al., 2014; Yoo et al., 2016). We found that overall, beta peak frequency increases over the first year of life and beyond, and beta burst rate decreases around the onset of movement in adults, but not until the hand contacts the object in 9- and 12-month-olds. We use dynamic time warping to show that infant beta bursts have the same waveform shape as adult bursts, differing only in that they are stretched in time. Principal component analysis (PCA) of burst waveforms was then used to identify common waveform motifs that are increasingly rate-modulated, earlier during the movement, and in an increasingly lateralized way, from infancy to adulthood. These results highlight the importance of considering the diverse features and potential functions of beta bursts rather than conceptualizing beta as a single phenomenon, and suggest that changes in burst rate and waveform motifs reflect developmental changes in the contralateral organization of the motor system and support the emergence of internal models for motor preparation and planning.

## Materials and Methods

### Participants

We analyzed data previously collected from 44 full-term 9-month-old infants (25 female, age range 8.6-9.93 months), 46 full-term 12-month-old infants (26 female, age range 11.2-12.93 months), and 23 adults (10 females, age range 18-22 years) recruited for a study on the neural bases of action execution and observation (Cannon et al., 2014; Yoo et al., 2016). Sixteen 9-month-old infants were excluded because of unusable EEG data before preprocessing (*N* = 5), fussiness after applying the EEG electrode not (*N* = 6), and never grasping the toy during the experiment (*N* = 1). Thirteen 12-month-old infants were excluded because of unusable EEG data before preprocessing (*N* = 6), becoming distressed shortly after applying the EEG electrode net (*N* = 6), and recording failure (*N* = 1). One adult participant was excluded because of a data recording error. The final sample therefore included 28 9-month-old infants, 33 12-month-old infants, and 22 adults. All infants were typically developing with no known or suspected neurodevelopmental or medical diagnoses. Before an infant's participation in the study, informed consent was obtained from the infant's parents. All adults had normal or corrected-to-normal vision and did not have any neurologic disorder. They provided informed consent before participating in the study. The experiment was approved by the University of Maryland Institutional Review Board.

### Procedure and task

Both infants and adult participants performed the same task. The infants were seated on their caregiver's lap, whereas the adults were seated in a chair, ∼40 cm away from a black puppet stage (99 cm × 61 cm × 84 cm), which was placed on a table. The area surrounding the stage was covered by black curtains to conceal the experimenter and any equipment from the participant's view. A video camera was placed behind the presenter to record any significant behavioral events that occurred during the testing, and the caregivers of the infants were told to remain passive.

The task comprised two conditions: observation and execution. To start the observation trials, the curtain was raised, revealing a female presenter. The presenter made eye contact with the participant and then looked toward a toy that was positioned in the center of the stage, but not within the participant's reach. The presenter then picked up the toy using a hand-operated claw-like tool, brought it to themselves, and briefly shook it. The curtain was lowered to mark the end of this trial, which lasted ∼4 s. For the execution trials, a toy was placed on the table; and while the presenter was hidden from the participant's view, the table was pushed toward the participant within reach as the curtain was raised. Participants were given ∼60 s to reach for the toy. The table was then pulled back and the curtain was lowered to mark the end of this trial. The order of the observation and execution trials was pseudo-randomized.

Ten different toys were utilized, with each toy being used in two observation and two execution trials. For adults, there was a maximum of 20 trials per condition per adult. On average, 9-month-old infants each completed 14.21 trials (SD = 4.82), 12-month-old infants completed 13.30 trials (SD = 4.82), and all adults completed 19.64 trials (SD = 1.71) per condition.

### Behavioral coding

Behavioral events were captured using video recorded at a resolution of 640 × 480 pixels and 30 Hz frame rate, and synchronized with the EEG recording. Two coders viewed the video offline and identified the frames when various events occurred (100% overlap). For the execution condition, these events were the first touch of the toy with the hand, and the completion of the grasp. The same events were coded for the observation condition, but the first touch was the time when the presenter first touched the toy with the tool, and grasp completion was the completion of the grasp of the toy with the tool. For the execution condition, the hand or hands used to grasp the toy were also coded (in the observation condition, the presenter always used their right hand). The coders achieved an inter-rater agreement of 84%-94% within a three-frame window of ∼100 ms (9m execution: first touch 93%, grasp complete 89%; 9m observation: first touch 92%, grasp complete 92%; 12m execution: first touch 93%, grasp complete 86%; 12m observation: first touch 91%, grasp complete 94%; adult execution: first touch 84%, grasp complete 94%; adult observation: first touch 93%, grasp complete 94%). These coded time points, as well as the start and end of the trials, were then used to segment the EEG data into epochs centered on these events.

### EEG recording and preprocessing

EEG was recorded using a 65-channel HydroCel Geodesic Sensor Net (Electrical Geodesics). The vertex (Cz) electrode was used as an online reference. EEG data were sampled at 500 Hz using EGI's Net Station (version 4.5.4) software. Impedances were kept <100 kΩ. After recording, EEG data were exported to a MATLAB compatible format using NetStation software for offline processing.

Both infant and adult datasets were preprocessed using MATLAB R2018a with a custom version of the MADE pipeline (Debnath et al., 2020), which was modified to include artifact detection routines from the NEAR pipeline (Kumaravel et al., 2022). The data were high pass filtered at 1 Hz and low pass filtered at 100 Hz using EEGLAB version 14.1.1 (Delorme and Makeig, 2004) FIR filters. Artifact-laden channels were identified and removed using the local outlier factor metric from the NEAR pipeline with an adaptive threshold starting at 2.5 for outlier detection (9m infants: 0-13 channels, mean = 2, SD = 2.83; 12m infants: 0-8 channels, mean = 1.58, SD = 1.85; adults: 0-5 channels, mean = 1.18, SD = 1.59). Artifact subspace reconstruction from the NEAR pipeline was then used to identify and correct nonstereotyped artifacts, using a cutoff parameter, *k* = 13. Stereotyped artifacts, such as eye blinks, eye movements, and data discontinuities, were then detected and removed using the independent component analysis (ICA)-based techniques from the MADE pipeline. ICA was performed on an identical copy of the dataset, which was first segmented into 1 s epochs. Noisy epochs in the copied dataset were removed using a voltage threshold of ±1000 μV. After ICA decomposition, independent components (ICs) were transferred from the copied dataset to the original dataset, which was used from then on. Artifactual ICs were removed from the original dataset using the EEGLAB Adjusted-ADJUST plugin, using default values for blink identification thresholds and α peak detection parameters (Mognon et al., 2011; Leach et al., 2020) (9m infants: 3-50 components, mean = 18.14, SD = 11.67; 12m infants: 6-52 components, mean = 18.48, SD = 10.22; adults: 6-34 components, mean = 16.77, SD = 6.98). The data were then divided into 3.6-s-long epochs, centered on four events: the start of the trial, the moment the toy was first touched, the completion of the grasp, and the end of the trial. A voltage threshold of ±150 µV was used to detect artifacts in each channel during each epoch. For each epoch, if >10% of the channels contained artifacts, the epoch was removed (9m infants: 0-30 epochs, mean = 5.68, SD = 7.95; 12m infants: 0-50 epochs, mean = 5.94, SD = 11.79; adults: no epochs removed); otherwise, the artifacted channels were removed and interpolated for that epoch. After artifact rejection, any remaining missing channels were interpolated, and the data were average rereferenced. Finally, line noise (60 Hz) was removed using an iterative version of the Zapline algorithm (de Cheveigné, 2020) implemented in the MEEGKit package (https://nbara.github.io/python-meegkit/), using a window size of 10 Hz for polynomial fitting and 2.5 Hz for noise peak removal and interpolation. For the execution condition, epochs during trials in which no grasp was made, the grasp used two hands, the toy was touched twice before grasping, or the grasp took longer than 1.6 s to complete were rejected. Following preprocessing, for each epoch, all participants with at least 5 trials were included in the following analyses (Table 1). All source code for preprocessing is available at https://github.com/danclab/dev_beta_claw/tree/main/preprocessing.

**Table 1.** Participants and trials used in analyses*^a^*

		9 months		12 months		Adults	
		*N*	Trials	*N*	Trials	*N*	Trials
Execution	Start	27	5-19, mean = 13.00, SD = 3.38	31	6-20, mean = 12.55, SD = 4.75	22	11-20, mean = 19.50, SD = 1.92
	Touch	22	5-18, mean = 10.09, SD = 3.26	25	5-18, mean = 10.36, SD = 3.51	22	10-20, mean = 19.45, SD = 2.13
	Grasp	22	5-18, mean = 10.14, SD = 3.41	25	5-18, mean = 10.40, SD = 3.45	22	10-20, mean = 19.45, SD = 2.13
	End	22	5-17, mean = 9.82, SD = 3.19	24	5-17, mean = 10.12, SD = 3.48	22	10-19, mean = 18.50, SD = 1.92
Observation	Start	28	5-19, mean = 13.57, SD = 4.34	32	5-19, mean = 12.72, SD = 4.31	22	11-20, mean = 18.68, SD = 1.73
	Touch	28	5-19, mean = 13.79, SD = 4.40	33	5-19, mean = 12.85, SD = 4.54	22	11-20, mean = 18.68, SD = 1.73
	Grasp	28	5-19, mean = 13.79, SD = 4.39	33	5-19, mean = 12.91, SD = 4.56	22	11-20, mean = 18.68, SD = 1.73
	End	27	8-19, mean = 13.74, SD = 4.05	33	5-19, mean = 12.55, SD = 4.44	22	10-20, mean = 18.64, SD = 1.94

*^a^*The number of subjects per age group with at least 5 trials, and the range, mean, and SD of the number of trials per subject for each epoch.

### Kinematics analysis

Before excluding trials in which two hands were used to grasp the toy, the type of grasp (unimanual or bimanual) was analyzed for the execution condition using a GLM model with a binomial distribution and logit link function with age as a fixed effect and subject-specific intercepts as random effects. The hand used (left or right) for unimanual movements in the execution was then analyzed in the same way. These were not analyzed for the observation condition because the adult actor always performed the action using their right hand. The time from the start of the trial until the first touch of the toy (reach duration), from the first touch until grasp completion (grasp duration), and from grasp completion until the end of the trial (manipulation duration) were analyzed separately for the execution (including only unimanual trials) and observation conditions using linear mixed models, including age as a fixed effect, and subject-specific intercepts as random effects. All analyses of movement kinematics were conducted using R (version 3.6.1) (R Core Team, 2022) and lme4 (version 1.1.29) (Bates et al., 2014). Fixed effects were assessed using Type II Wald χ^2^ tests (car version 3.1.0) (Fox et al., 2019). Pairwise Tukey-corrected follow-up tests were run using estimated marginal means from the emmeans package (version 1.7.3) (Lenth et al., 2020).

### Burst analyses

Power spectral densities were computed from 0.1 to 100 Hz with the MNE-Python toolbox (Gramfort et al., 2014) using Welch's method (Welch, 1967) with a window size of 1 s, 50% overlap, and 10 times oversampling, resulting in a frequency resolution of 0.1 Hz. For each participant (infants and adults), this was applied to all data (i.e., from both epochs of the observation and execution conditions). For each channel, the power spectral density was parameterized using FOOOF (Donoghue et al., 2020) to obtain estimates of the aperiodic and periodic spectral components. The periodic spectra were then averaged over all electrodes in the C3 and C4 clusters (E16, E20, E21, E22, E41, E49, E50, E51), and then across participants within each age group. Group-level spectral peaks in the periodic component were identified using an iterative procedure in which a Gaussian function was fitted to the global maximum and subtracted from the periodic spectral density. The procedure was then repeated using the result of the subtraction, continuing until there are no more peaks above the noise floor (1 SD across all frequencies). For each peak, the frequency band limits were determined by computing the FWHM of the peak power, and only bands <10 Hz with a FWHM of at least 1 Hz or >10 Hz with a FWHM of at least 3 Hz were retained.

Lagged coherence was computed for all data from each participant (e.g., from both the observation and execution conditions) from 5 to 100 Hz in 1 Hz increments and 2-4.5 cycles in increments of 0.1 cycles (Fransen et al., 2015) using FieldTrip version 20190329 (Oostenveld et al., 2011). We used overlapping epochs with lag- and frequency-dependent widths. Fourier coefficients were obtained for each epoch using a Hann-windowed Fourier transform. Because lagged coherence depends on data SNR (Fransen et al., 2015), we normalized lagged coherence values for each participant by the maximum lagged coherence over all channels, frequencies, and lags for that participant.

We used the superlet transform (Moca et al., 2021) to compute single-trial TF decompositions for each electrode with optimally balanced time and frequency resolution. We used an adaptive superlet transform based on Morlet wavelets with varying central frequency (1-100 Hz) and number of cycles (4 cycles) under a Gaussian envelope. The order was linearly varied from 1 to 40 over the frequency range.

For each channel of the C3 and C4 clusters, within the beta frequency band identified, we used an adaptive burst detection algorithm to detect all potentially relevant burst events across a wide range of beta amplitudes during the execution condition (Szul et al., 2022). Similar to the method used for peak detection with the power spectral densities, the algorithm first subtracts the estimated aperiodic spectrum from each single-trial TF decomposition and on each iteration, detects the global maximum amplitude in TF space and fits a two-dimensional Gaussian to this peak by computing the symmetric FWHM in the time and frequency dimensions. The Gaussian is then subtracted from the TF decomposition, and the next iteration operates on the resulting residual TF matrix. This process continues until there are no global maxima above the noise floor remaining (2 SDs above the mean amplitude over all time and frequency bins, recomputed on each iteration). We applied the algorithm to TF data within a window 5 Hz wider than the identified beta band on either side, but only bursts with a peak frequency within the band were retained for further analysis.

The waveform for each detected burst was extracted from the “raw” time series (only 1 Hz high pass and 100 Hz low pass filtered during preprocessing), based on their peak time. To eliminate the influence of slower ERP dynamics on burst waveforms, the epochs were first averaged in the temporal domain to calculate the ERP, and this was then removed from the signal for each trial. The width of the time window for waveform extraction was determined using the FWHM of lagged coherence averaged within the beta band. The time series within this window, centered on the peak time, was then extracted from the trial time series. To find the signal deflection corresponding to the peak in TF amplitude, the burst waveforms were aligned by band pass filtering them within their detected frequency span (using a zero-phase FIR filter with a Hamming window), calculating their instantaneous phase using the Hilbert transform, and recentering the “raw” waveform (before band pass filtering) around the phase minimum closest to the peak time detected in TF space (Boto et al., 2022; Szul et al., 2022). If this time point was >30 ms away from the TF-detected peak time, the burst was discarded. The DC offset was then subtracted from the resulting waveform. Finally, to account for uncertainty in the orientation and source location of the dipoles that generated the signals measured by the EEG electrodes, the sign of burst waveforms in which the central deflection was positive was reversed (Jones et al., 2009; Szul et al., 2022). Open-source code for the burst detection algorithm can be found at https://github.com/danclab/burst_detection.

Because infants tended to use either hand to grasp the toy, the time courses of burst rate and beta power in the execution condition were analyzed according to which electrode cluster (C3 or C4) was contralateral or ipsilateral to the hand used. In the observation condition, bursts were simply categorized according to the electrode cluster they were identified in (C3 or C4). The burst rate was computed by binning bursts in 25 ms time bins, and then smoothing the resulting histogram with a Gaussian kernel (width = 3 time points). Both burst rate and mean beta amplitude (averaged within the identified beta band) were baseline-corrected using the mean rate or amplitude during the 1.5 s before the start of the trial, and expressed as a percentage change from baseline. To identify significant deviations from the baseline, we used a one-sample cluster permutation test. The family-wise error rate was controlled using a nonparametric resampling test with a maximum statistic (taken across all data points). A *t* test with relative variance regularization (“hat” adjustment; σ = 0.001) (Ridgway et al., 2012) was used as the statistic to minimize the impact of low variance data points and prevent spurious results. Threshold-free cluster enhancement was used to enhance the statistical power of cluster detection by using an adaptive threshold on the level of a single data point (starting threshold = 0, step = 0.01) (Smith and Nichols, 2009).

Burst peak amplitude, peak frequency, frequency span, and duration were analyzed using R (version 3.6.1) (R Core Team, 2022) with linear mixed models, including age, or age, cluster, and their interactions as fixed effects, and subject-specific intercepts as random effects (lme4 version 1.1.29) (Bates et al., 2014). Because the peak frequency was necessarily different across ages because of the beta band identification procedure, the analysis of the effect of cluster on peak frequency included subject nested within age as random effects, and duration as analyzed in terms of cycles (computed as the burst duration in seconds divided by the burst peak frequency). Fixed effects were assessed using Type III Wald χ^2^ tests (car version 3.1.0) (Fox et al., 2019). Pairwise Tukey-corrected follow-up tests were run using estimated marginal means from the emmeans package (version 1.7.3) (Lenth et al., 2020).

Burst waveforms from 9- and 12-month-old infants were separately warped to adult burst waveforms using dynamic time warping (Giorgino, 2009). To account for differences in burst amplitude, we first normalized the median burst waveform for each group, and performed dynamic time warping on the normalized median infant burst waveforms using Rabiner-Juang step patterns (Type 5c) (Rabiner and Juang, 1993), with the normalized median adult burst waveform as the reference. The resulting alignments were then used to warp all infant bust waveforms.

To classify burst waveform shapes, PCA (20 components, implemented in the scikit-learn library) (Pedregosa et al., 2011) was applied to the warped burst waveforms from all age groups (Szul et al., 2022). All detected bursts were then projected onto each principal component (PC), with each burst thus having a score for each component representing the shape of its waveform along that dimension. To determine which components were not simply caused by noisy signal fluctuations, we used a permutation approach (Vieira, 2012) to remove correlations between features (waveform time points). The matrix containing all burst waveforms was shuffled within each time point (column) independently, and PCA was applied to the shuffled matrix. The *p* value for each PC was then given by the probability of the proportion of variance explained being lower after shuffling than that for the unshuffled data. One hundred permutations were run for each component, with an α threshold of *p* = 0.0035, using Bonferroni correction for multiple comparisons. To evaluate consistency in PCA results across age groups, we ran a separate PCA for each age group, using only bursts detected from participants in that group. For each group PCA, we examined correlations between its eigenvectors and those of the global PCA by constructing a correlation matrix comparing each component from one PCA to each component of the other. Because the direction of each dimension identified by PCA is arbitrary, we took the absolute value of the correlation coefficient. To account for different potential ordering of components, we looked at the maximum correlation in each row of the matrix (i.e., for each component of the global PCA, the component of the group PCA most correlated with it). This same procedure was used to examine correlations between burst scores after projecting their waveforms onto each dimension of the global and group PCAs.

We proceeded to select PCs that define dimensions along which the mean burst waveform shape varied during either the action observation or execution conditions. For each PC, the mean burst waveform score was computed for each epoch in 50 ms bins over both electrode clusters. Subsequently, the scores were smoothed using a Gaussian kernel with a width of 2 time points and baseline-corrected using the 1.5 s preceding the beginning of the trial. To ascertain when the mean score deviated from the baseline for each PC, a one-sample cluster permutation test was used over all subjects from all age groups. To control the family-wise error rate, a nonparametric resampling test with a maximum statistic (taken across all data points) was used. A *t* test with a relative variance regularization (i.e., “hat” adjustment) was used, and the threshold for significance was set at *p* = 0.000025, Bonferroni-adjusted for multiple comparisons. Threshold-free cluster enhancement was used, starting with a threshold of 0 and a step size of 0.2. Components with mean scores that significantly deviated from baseline during the toy touch, grasp completion, and trial end epochs in either the action observation or execution conditions were selected for further analysis.

To analyze the burst rate according to waveform shape, we binned bursts according to their component score, indicating their waveform shape (four quartiles), and the time during the trial in which they occurred (50 ms bins). For each component score quartile, we then baseline-corrected the burst rate as described above, but using only bursts with a component score within that quartile in order account for quartile-specific differences in baseline rate, and smoothed it using a Gaussian kernel (width = 2 time points). We used one-sample cluster permutation tests to determine when burst rates in each component quartile significantly deviated from the baseline. The family-wise error rate was controlled using a nonparametric resampling test with a maximum statistic (taken across all data points). A *t* test with a relative variance regularization (“hat” adjustment; threshold *p* = 0.0000156, Bonferroni-adjusted for multiple comparisons) and threshold-free cluster enhancement (starting threshold = 0, step = 0.2) was used.

All source code for these analyses is available at https://github.com/danclab/dev_beta_claw.

## Results

### Behavioral kinematics

Overall, the kinematics analysis revealed several significant differences in the behavior of participants of different age groups. The great majority of movements in the execution condition were unimanual, and there was no difference in the amount of bimanual movements between age groups (χ^2^(2) = 5.57, *p* = 0.062; 9m mean = 0, SD = 0% of trials; 12m mean = 7.53, SD = 26.42% of trials; adult mean = 1.40, SD = 11.74% of trials). There was a difference in the hand used for unimanual movements (χ^2^(2) = 21.06, *p* < 0.001), with adults using the right hand (mean = 84.62, SD = 36.12% of trials) more than 9-month-olds (*Z* = 4.00, *p* < 0.001; 9m mean = 60.33, SD = 48.99% of trials) and 12-month-olds (*Z* = 4.37, *p* < 0.001; 12m mean = 60.15, SD = 49.02% of trials), but there was no difference between 9- and 12-month-olds (Z = −0.35, *p* = 0.935).

In the execution condition, the time until the first touch of the toy was significantly different between the groups (χ^2^(2) = 25.15, *p* < 0.001), with adults reaching the toy faster (mean = 1.71, SD = 0.84 s) than both 9-month-olds (*t*_(72.6)_ = −3.55, *p* = 0.002; 9m mean = 8.00, SD = 13.97 s) and 12-month-olds (*t*_(74.1)_ = −4.90, *p* < 0.001; 12m mean = 9.63, SD = 16.81 s). There was no significant difference in the time to reach between 9- and 12-month-olds (*t*_(84.8)_ = 1.30, *p* = 0.401). The duration of the grasping (χ^2^(2) = 232.16, *p* < 0.001) and manipulation movements (χ^2^(2) = 30.65, *p* < 0.001) were also significantly different between the groups, with adults performing faster grasping (mean = 0.25, SD = 0.16 s) and manipulation movements (mean = 1.41, SD = 0.40 s) faster than both 9-month-olds (grasping: *t*_(69.9)_ = −13.74, *p* < 0.001; 9m mean = 0.73, SD = 0.33 s; manipulation: *t*_(67.6)_ = −5.19, *p* < 0.001; 9m mean = 2.49, SD = 3.28 s) and 12-month-olds (grasping: *t*_(71.7)_ = −12.81, *p* < 0.001; 12m mean = 0.68, SD = 0.35 s; manipulation: *t*_(69.5)_ = −4.29, *p* < 0.001; 12m mean = 2.33, SD = 1.96 s). However, there was no significant difference in grasp duration (*t*_(86.7)_ = −1.29, *p* = 0.403) or manipulation time (*t*_(88.7)_ = −1.01, *p* = 0.574) between 9- and 12-month-olds. In summary, there were no differences in the kinematics of performed actions between 9 and 12 months, but adults performed faster reach, grasp, and manipulations movements than both infant age groups.

During the observation task, there was no significant difference between age groups in the duration of the observed reach (χ^2^(2) = 0.49, *p* = 0.782; 9m mean = 1.89, SD = 2.34 s; 12m mean = 1.87, SD = 0.75 s; adult mean = 1.81, SD = 0.92 s). However, the durations of the observed grasp (χ^2^(2) = 11.83, *p* = 0.003) and manipulation movements (χ^2^(2) = 7.45, *p* = 0.024) were significantly different, with 12-month-olds observing longer grasps (*t*_(77.4)_ = −3.33, *p* = 0.004; 12m mean = 0.68, SD = 0.31 s; adult mean = 0.53, SD = 0.28 s) and manipulation movements (*t*_(79.5)_ = 2.57, *p* = 0.032; 12m mean = 2.76, SD = 0.38 s; adult mean = 2.55, SD = 0.33 s). However, there were no significant differences in grasp or manipulation duration during observation between 9-month-olds and either 12-month-olds (grasp: *t*_(82.0)_ = 2.23, *p* = 0.071; 9m mean = 0.58, SD = 0.29 s; manipulation: *t*_(80.4)_ = 0.28, *p* = 0.958; 9m mean = 2.77, SD = 0.35 s) or adults (grasp: *t*_(76.6)_ = −1.18, *p* = 0.472; manipulation: *t*_(79.3)_ = −2.23, *p* = 0.072). There were therefore no consistent differences in the observed action kinematics with age.

### Sensorimotor beta occurs as transient bursts in infancy and adulthood

**Figure 1.**
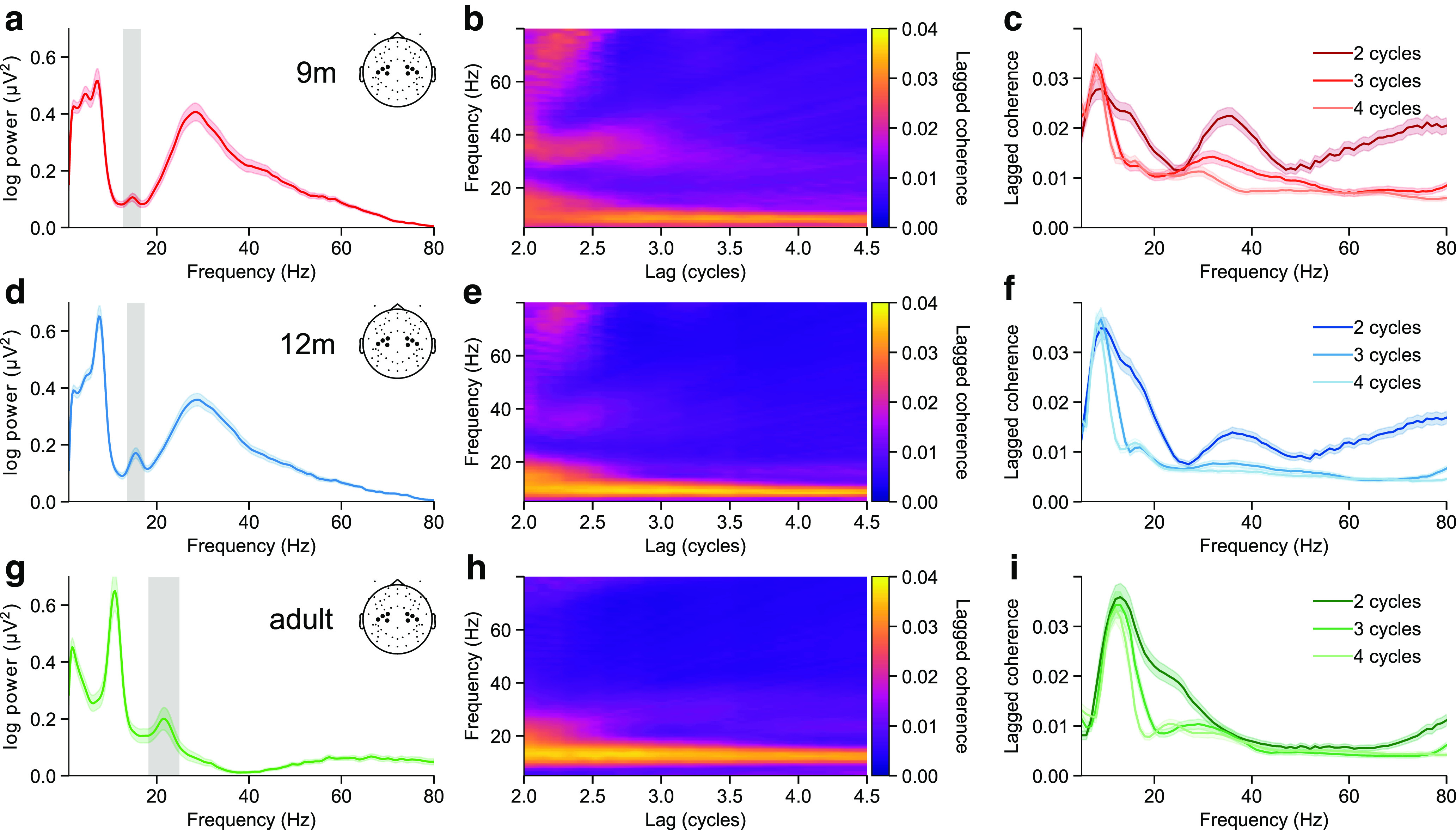
Beta peak frequency increases with age, and beta activity consists of transient burst events in infancy and adulthood. ***a***, Periodic power spectral density in the combined C3 and C4 cluster of the 9-month-old participants. Dark lines indicate the mean over participants. Shaded areas represent SE. Gray shaded region represents the limits of the identified beta band. Inset, The electrodes included in the analysis. ***b***, Mean lagged coherence in the combined C3 and C4 cluster across all 9-month-old participants for a range of frequencies and lags. There is high lagged coherence in the alpha band over a wide range of lags, but beta lagged coherence rapidly decreases after 2 cycles. ***c***, Mean lagged coherence in the combined C3 and C4 cluster across all 9-month-old participants for lags of 2, 3, and 4 cycles. Solid lines indicate the mean. Shaded areas represent SE. A prominent peak appears in the α range at 2-4 cycles, while a beta peak is visible only at 2 cycles. ***d-f***, Same as in ***a-c***, for 12-month-old participants. ***g-i***, Same as in ***a-c***, for adult participants.

We first sought to confirm whether sensorimotor beta activity is “bursty” rather than oscillatory in infants and adults. With this aim, we analyzed EEG data recorded from clusters of electrodes centered around C3 and C4, while 9-month-old, 12-month-old, and adult participants reached for, grasped, and shook a toy, or observed an adult performing the same series of actions. After decomposing the power spectrum within these clusters into aperiodic and periodic components (Donoghue et al., 2020; Ostlund et al., 2022), we identified group-level periodic peaks within the canonical beta frequency range (13-30 Hz) in each age group from the periodic spectra averaged over all electrodes, conditions, and epochs. The identified beta frequency bands increased in peak frequency and range from 9 months to adulthood (9 months: 12.75-16.25 Hz, 12 months: 13.5-17.0 Hz, adults: 18.25-24.75 Hz, Fig. 1*a*,*d*,*g*) (Meyer et al., 2016; Rayson et al., 2022). The periodic spectrum peak with a peak frequency just below the beta peak was identified as α/μ (9 months: 5.5-9.0 Hz, 12 months: 5.75-9.25 Hz, adults: 9.25-12.75 Hz).

We evaluated the rhythmicity of activity across the entire frequency spectrum using lagged coherence from 2 to 4.5 cycles in increments of 0.1 cycles. Activity in the α/μ frequency range had a high lagged coherence value that was sustained over at least 4.5 cycles, but lagged coherence in the beta frequency bands fell rapidly after 2 cycles (Fig. 1*b*,*e*,*h*), meaning that its phase was unpredictable after 2 cycles in the future. This indicates that sensorimotor α/μ activity occurred as a rhythmic oscillation sustained over several cycles, whereas beta activity occurred as transient bursts (Fig. 1*c*,*f*,*i*).

**Figure 2.**
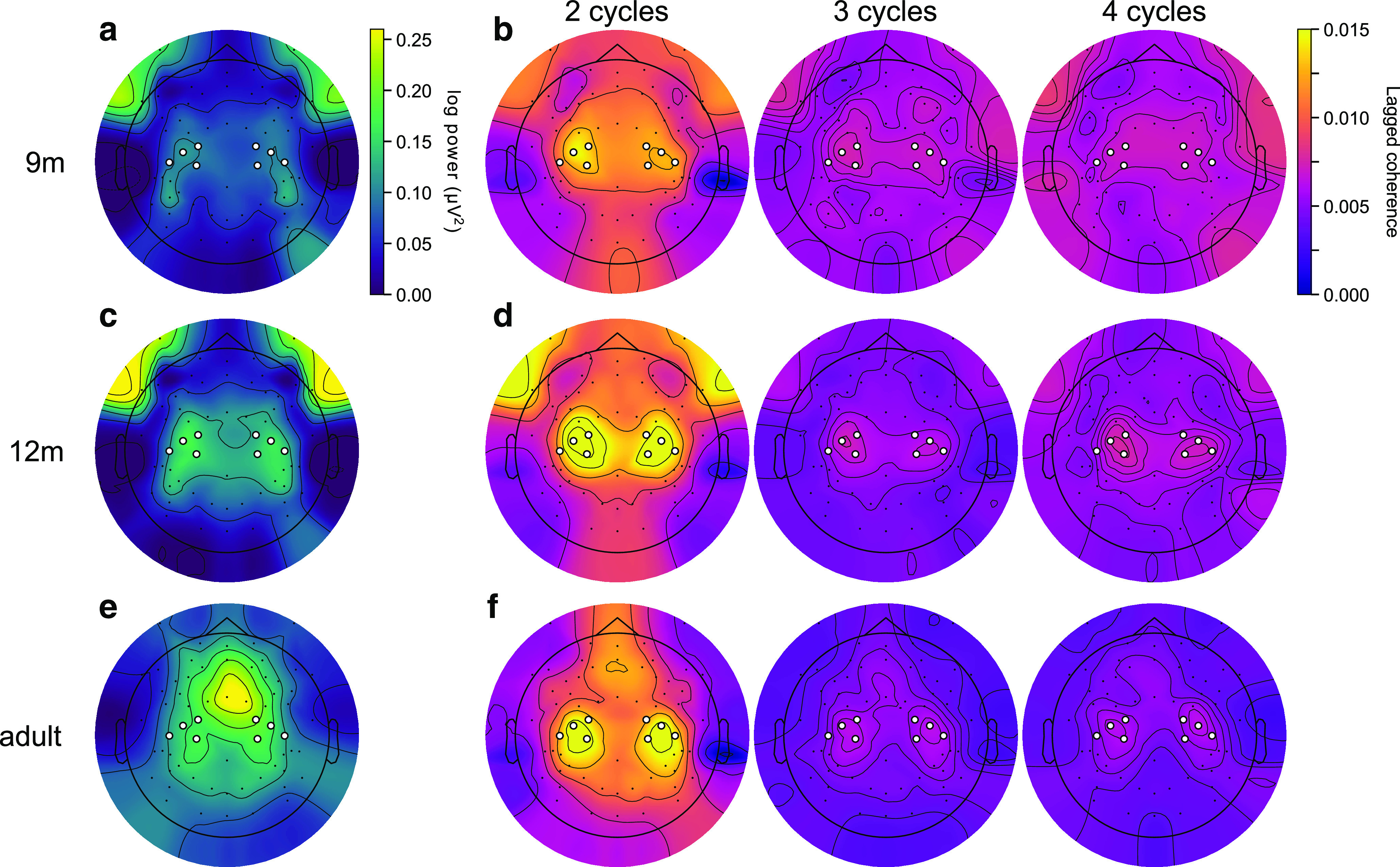
Power and lagged coherence localize beta to the C3 and C4 clusters. ***a***, Topography of beta band periodic power (after subtraction of the aperiodic spectral density), averaged over 9-month-old participants. White circles represent electrodes included in the C3 and C4 clusters. Power in the beta band is most prominent in peripheral electrodes. ***b***, Beta lagged coherence topographies at (from left to right) 2, 3, and 4 cycles averaged over 9-month-old participants. Lagged coherence in the beta band localizes to the C3 and C4 electrodes and decreases rapidly after two cycles. ***c***, ***d***, Same as in ***a***, ***b***, for the 12-month-old participants. ***e***, ***f***, Same as in ***a***, ***b***, for the adult participants.

**Figure 3.**
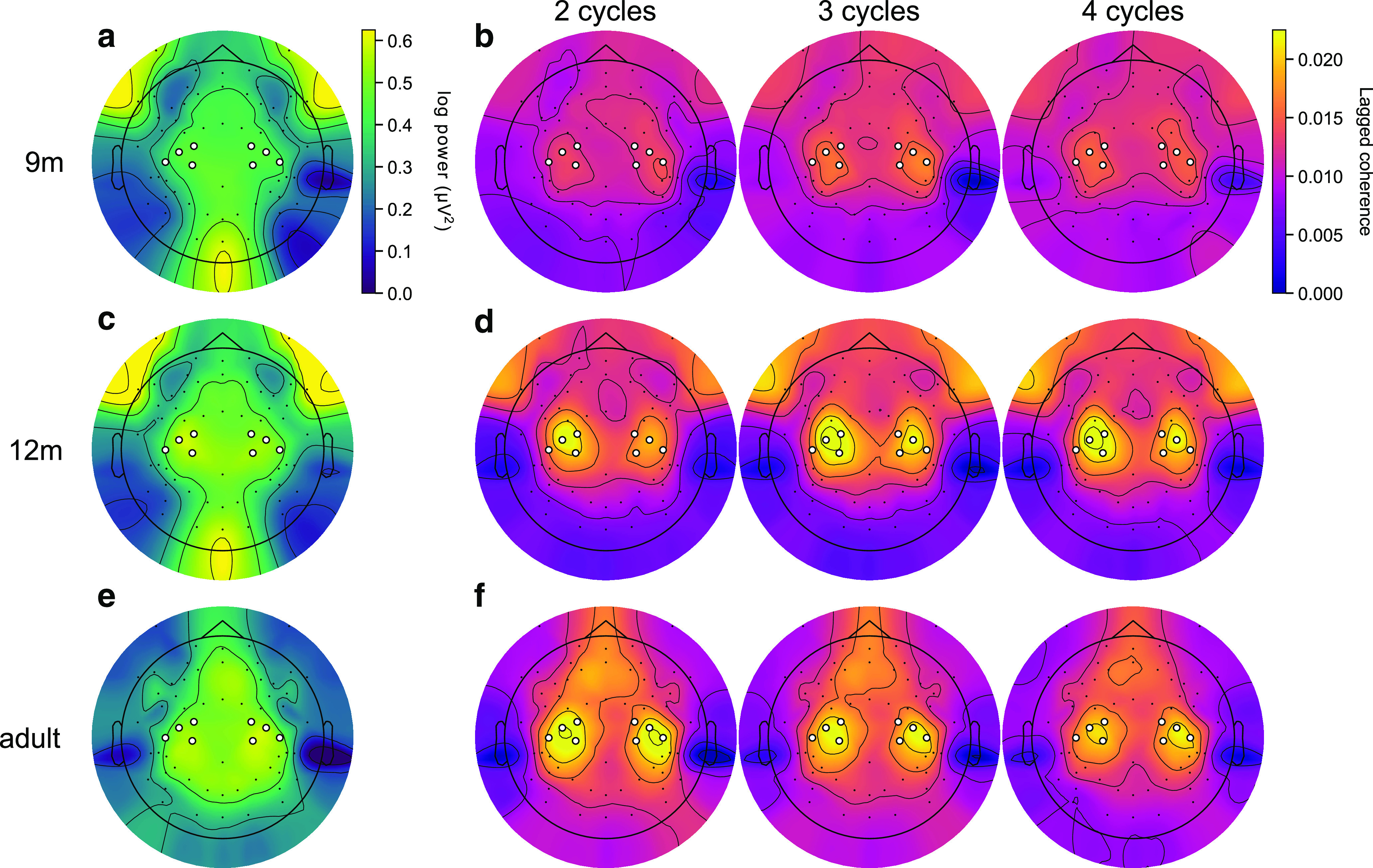
***a-f***, Alpha/μ occurs as a sustained oscillation in the C3 and C4 clusters. Same as in Figure 2, for the α/μ band. Power in the α/μ band is strongest in peripheral, central, and occipital electrodes in infants, and in central and frontal electrodes in adults. Lagged coherence localizes α/μ to the C3 and C4 electrodes and remains high up to at least 4 cycles at all ages.

Beta power and lagged coherence had different spatial topographies across age groups. Beta power localized to peripheral locations in 9- and 12-month-old infants (Fig. 2*a*,*c*), but to the C3 and C4 clusters and central frontal electrodes in adults (Fig. 2*e*). However, in all age groups, lagged coherence in the beta band localized to the C3 and C4 electrodes at 2 lag cycles, and rapidly decreased with increasing cycles (Fig. 2*b*,*d*,*f*). Alpha/μ activity had a very different pattern of power and lagged coherence topographies (Fig. 3). The beta band therefore has distinct spectral and spatial signatures in infancy and adulthood, and the spatial topography of beta power and lagged coherence confirmed our choice of electrode clusters.

### Beta bursts have diverse time-frequency based features

**Figure 4.**
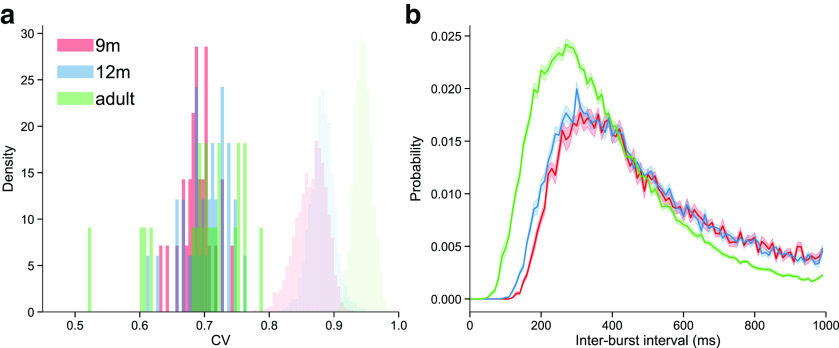
Beta burst timing is not a pure Poisson process. ***a***, The distribution of the coefficient of variation of the IBIs over participants from each age group (dark shaded histograms), and from surrogate data with random burst times (light shaded histograms). ***b***, The mean IBI distribution for each age group (solid lines; shaded area represents the SE).

To detect beta bursts, we used an adaptive, trial-based method to identify all potential burst events within each beta frequency range for each age group (Szul et al., 2022). We first examined the interburst intervals (IBIs) for each participant, and compared the coefficient of variation to that computed for surrogate data obtained by 1000 iterations of assigning each burst a random peak time during the trial. The coefficient of variation was <1, and lower than the 95% lower CI computed from the surrogate data for all participants from all age groups (9m: 0.63-0.74, mean = 0.69, SD = 0.02; 12m: 0.61-0.76, mean = 0.70, SD = 0.03; adult: 0.52-0.79, mean = 0.70, SD = 0.06; Fig. 4*a*), indicating that bursts occur more regularly than would be expected by a purely Poisson process. The IBI distribution revealed that the low coefficient of variation is the result of a time window just after a burst during which a consecutive burst is unlikely to occur (Fig. 4*b*), indicating that there is little temporal overlap in the bursts detected using this method.

**Figure 5.**
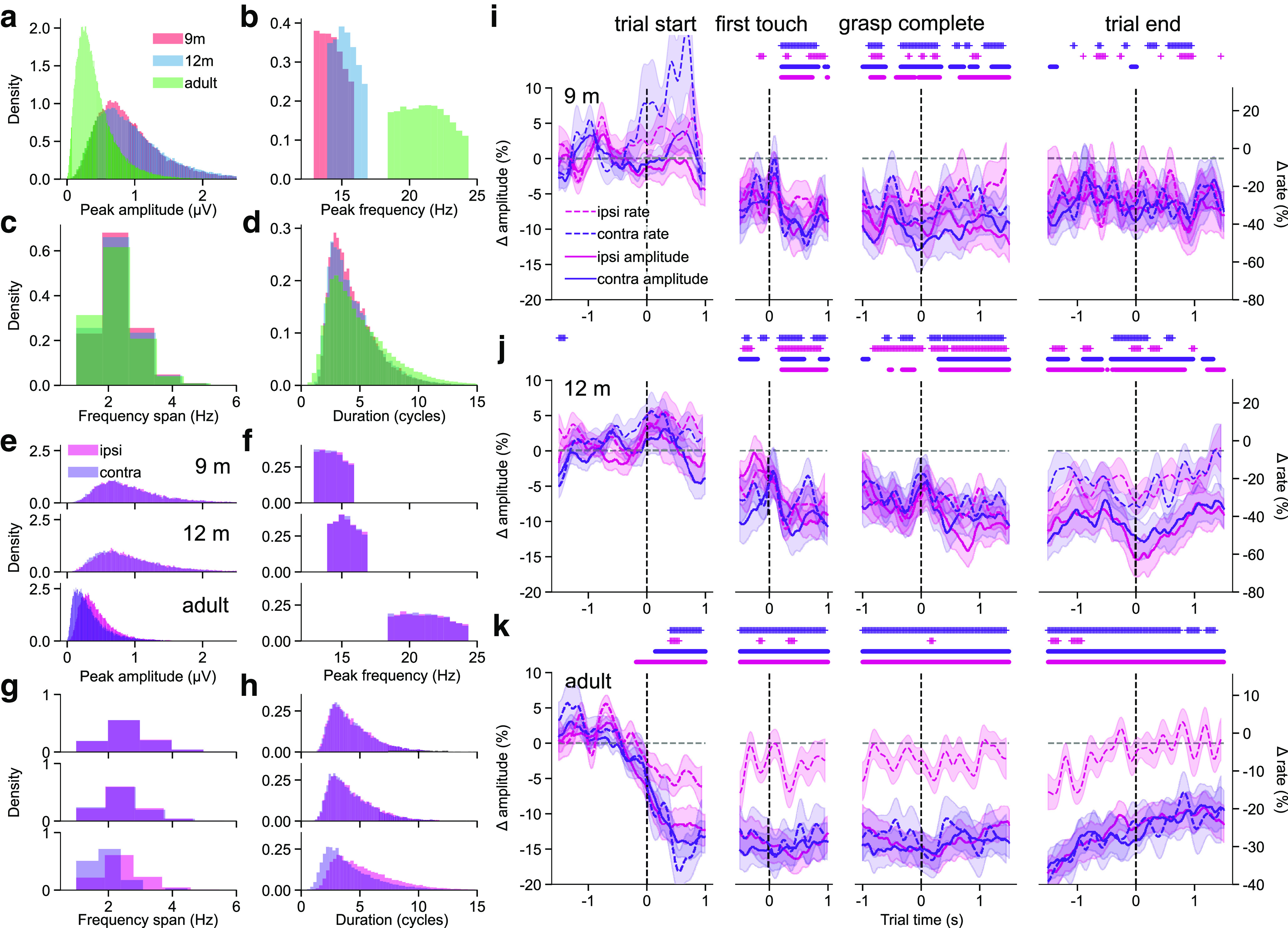
Beta bursts are diverse in infancy and adulthood. ***a-d***, The distributions of beta burst peak amplitude (***a***), peak frequency (***b***), frequency span (***c***), and duration (***d***) for the 9-month-old, 12-month-old, and adult participants over conditions, epochs, and clusters. ***e-h***, The distributions of burst peak amplitude (***e***), peak frequency (***f***), frequency span (***g***), and duration (***h***) for the (rows, from top to bottom), 9-month-old, 12-month-old, and adult participants for the ipsilateral and contralateral electrode clusters. ***i***, The mean burst rate (dashed lines; shaded area represents SE) and mean beta amplitude (solid lines; shaded area represents SE) in the ipsilateral and contralateral clusters for the 9-month-old participants in the execution condition. Colored dots and asterisks represent times in which beta amplitude or burst rate significantly deviated from baseline. ***j***, Same as in ***i***, for the 12-month-old participants. ***k***, Same as in ***i***, for the adult participants.

We then compared time-frequency based features of these bursts between age groups. Bursts across all age groups exhibited a wide range of peak amplitudes (Fig. 5*a*), frequencies (Fig. 5*b*), frequency spans (Fig. 5*c*), and durations (Fig. 5*d*). There was a main effect of age for burst peak amplitude (χ^2^(2) = 118.75, *p* < 0.001), frequency span (χ^2^(2) = 31.19, *p* < 0.001), and duration (χ^2^(2) = 25.41, *p* < 0.001). There was no difference in peak amplitude between 9- and 12- month-olds (*Z* = −0.21, *p* = 0.975; 9m mean = 0.97, SD = 0.63 μV; 12m mean = 0.99, SD = 0.66 μV), but adult bursts had lower peak amplitudes than those detected in 9-month-old (*Z* = −9.61, *p* < 0.001; adult mean = 0.42, *SD* = 0.30 μV) and 12-month-old infants (*Z* = −9.75, *p* < 0.001). Bursts detected in 12-month-olds had a slightly narrower frequency span as those detected in 9-month-olds (*Z* = −2.35, *p* = 0.049; 9m: mean = 2.12, SD = 0.82 Hz; 12m: mean = 2.08, SD = 0.82 Hz), and adult bursts were narrower than those of 9-month-olds (*Z* = −5.57, *p* < 0.001; adult: mean = 2.00, SD = 0.82 Hz) and 12-month-olds (*Z* = −3.56, *p* = 0.001). Finally, beta bursts were similar in duration at 12 months compared with at 9 months (*Z* = 0.13, *p* = 0.990; 9m: mean = 4.42, SD = 2.03 cycles; 12m: mean = 4.45, SD = 2.19 cycles), but lasted more cycles in adults compared with 9-month-olds (*Z* = 4.46, *p* < 0.001; adult: mean = 4.99, SD = 2.80 cycles) and 12-month-olds (*Z* = 4.49, *p* < 0.001). In summary, while bursts did not differ in peak amplitude or duration between 9- and 12-month-olds, they decreased in amplitude, decreased in frequency span, and increased in duration from infancy to adulthood.

We then sought to determine whether there was any movement-related lateralization in burst TF-based features and how this might change with age (Fig. 5*e–h*). We categorized bursts in the execution condition based on whether the electrode cluster they were detected from was contralateral or ipsilateral to the performed movement. Our results revealed an age-cluster interaction for burst peak amplitude (χ^2^(2) = 379.91, *p* < 0.001), peak frequency (after accounting for age-specific frequency ranges; χ^2^(2) = 12.54, *p* = 0.002), frequency span (χ^2^(2) = 1203.56, *p* < 0.001), and duration (χ^2^(2) = 2184.24, *p* < 0.001). At 9 months, there was no difference in burst peak amplitude between hemispheres (*Z* = −0.09, *p* = 0.927; contralateral: mean = 0.98, SD = 0.67 μV; ipsilateral: mean = 0.99, SD = 0.63 μV). However, at 12 months and in adults, contralateral bursts had lower amplitudes than ipsilateral bursts (12 months: *Z* = −4.92, *p* < 0.001; contralateral: mean = 0.97, SD = 0.72 μV; ipsilateral: mean = 1.00, SD = 0.66 μV; adults: *Z* = −35.31, *p* < 0.001; contralateral: mean = 0.35, SD = 0.29 μV; ipsilateral: mean = 0.44, SD = 0.27 μV). Contralateral and ipsilateral bursts had the same peak frequency at 9 months (*Z* = −1.23, *p* = 0.217; contralateral: mean = 14.31, SD = 0.97 Hz; ipsilateral: mean = 14.33, SD = 0.97 Hz) and 12 months (*Z* = −0.50, *p* = 0.616; contralateral: mean = 15.32, SD = 0.95 Hz; ipsilateral: mean = 15.33, SD = 0.96 Hz), but in adults, contralateral bursts had a lower peak frequency than ipsilateral bursts (*Z* = −7.05, *p* < 0.001; contralateral: mean = 21.12, SD = 1.77 Hz; ipsilateral: mean = 21.15, SD = 1.78 Hz). There was no difference in frequency span between hemispheres at 9 months (*Z* = −1.13, *p* = 0.258; contralateral: mean = 2.12, SD = 0.83 Hz; ipsilateral: mean = 2.13, SD = 0.82 Hz), but contralateral bursts had a narrower frequency span than ipsilateral bursts at 12 months (*Z* = −5.72, *p* < 0.001; contralateral: mean = 2.06, SD = 0.83 Hz; ipsilateral: mean = 2.11, SD = 0.84 Hz) and in adults (*Z* = −61.38, *p* < 0.001; contralateral: mean = 1.82, SD = 0.76 Hz; ipsilateral: mean = 2.12, SD = 0.83 Hz). Finally, ipsilateral and contralateral bursts did not differ in terms of duration at 9 months (*Z* = −1.18, *p* = 0.239; contralateral: mean = 4.39, SD = 2.05 cycles; ipsilateral: mean = 4.43, SD = 2.03 cycles), but contralateral bursts were shorter than ipsilateral bursts in 12-month-olds (*Z* = −5.15, *p* < 0.001; contralateral: mean = 4.31, SD = 2.07 cycles; ipsilateral: mean = 4.45, SD = 2.13 cycles) and in adults (*Z* = −80.19, *p* < 0.001; contralateral: mean = 4.20, SD = 2.42 cycles; ipsilateral: mean = 5.41, SD = 2.80 cycles). Contralateral and ipsilateral beta bursts did not differ in terms of TF-based features at 9 months, but by 12 months, contralateral bursts had a lower peak amplitude, narrower frequency span, and shorter duration than ipsilateral bursts, and by adulthood, contralateral bursts additionally had a lower peak frequency than ipsilateral bursts.

As found in previous studies of movement-related sensorimotor beta activity, changes in the overall burst rate generally tracked changes in mean beta amplitude in the execution condition (Little et al., 2019; Rayson et al., 2022) (Fig. 5*i–k*). In 9- and 12-month-old infants, there was a bilateral decrease in both the burst rate and mean beta amplitude following the first contact of the hand with the toy (Fig. 5*i*,*j*). However, in adults, the mean beta amplitude decreased bilaterally following the onset of the movement, whereas the burst rate only decreased contralaterally (Fig. 5*k*). Thus, while mean beta amplitude was bilaterally modulated by action execution in all age groups, only changes in the overall beta burst rate were lateralized, and only in adults. Beta activity was only modulated during action observation in adults around the time of the first contact between the hand and toy, and bilaterally for the mean amplitude, whereas the overall burst rate only decreased in C3.

### Infant beta bursts have the same mean waveform shape as adult bursts

**Figure 6.**
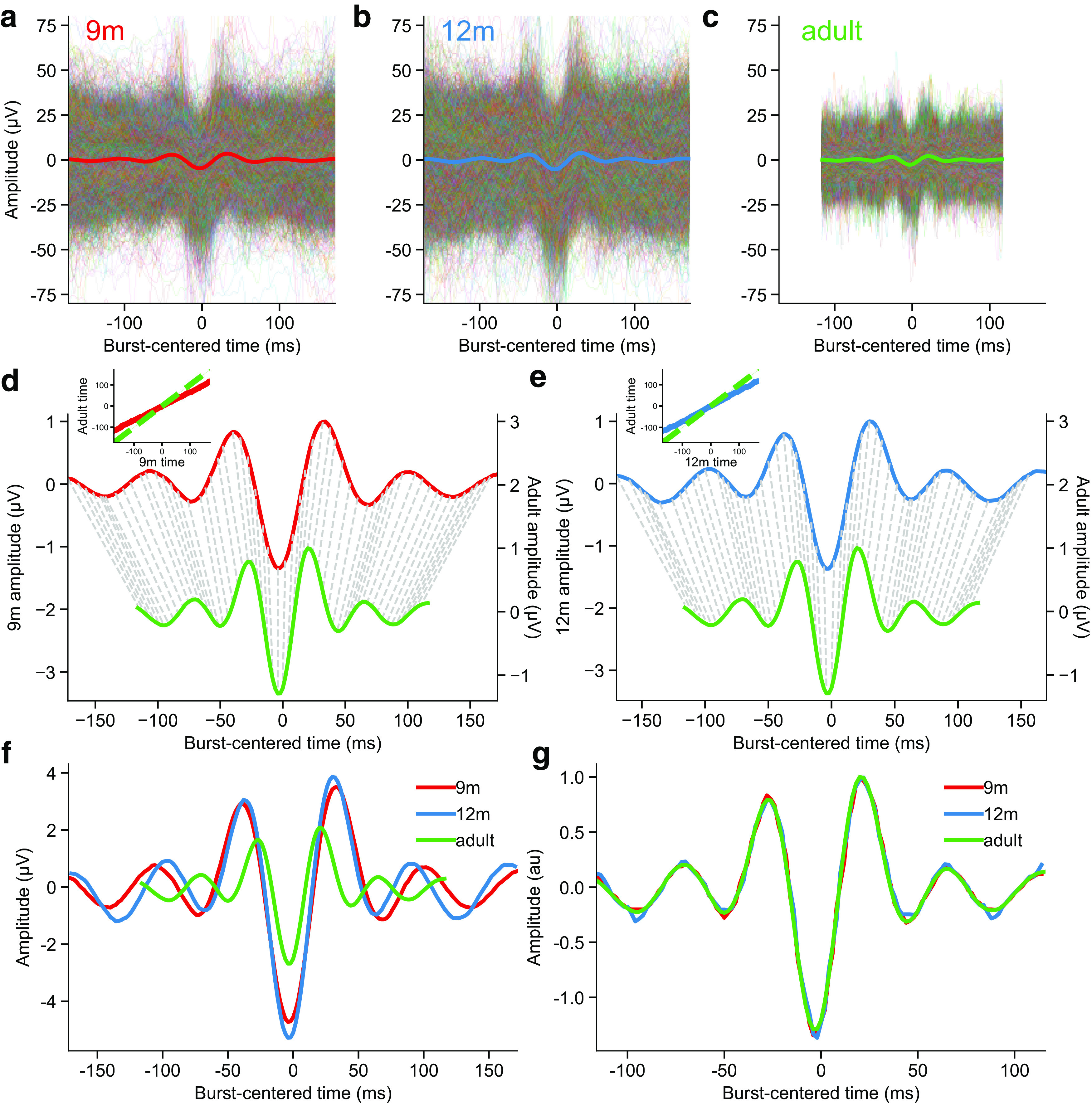
Infant and adult bursts have qualitatively similar burst waveforms. ***a***, The median waveform (thick red line) over all detected beta bursts from 9-month-old participants had a wavelet-like shape, but there was great variability in the waveforms of individual bursts (thin colored lines). ***b***, Same as in ***a***, for the 12-month-old participants. ***c***, Same as in ***a***, for the adult participants. ***d***, Correspondence between time points (dashed lines) of 9-month-old beta burst waveforms (red) and adult beta burst waveforms (green). Inset, The alignment curve (solid line) resulting from dynamic time warping of beta burst waveforms from 9-month-old participants to those of adults. The dashed line indicates the alignment curve for two already aligned signals. ***e***, Same as in ***d***, for the 12-month-old participants. ***f***, The adult beta burst waveforms were, on average, shorter in absolute duration and smaller in amplitude than the mean infant bursts. ***g***, Normalization and dynamic time warping revealed that the infant and adult beta bursts have qualitatively similar waveform shapes.

The differences in TF-based beta burst features between infants and adults give little insight into the mechanisms underlying these changes. All of these features are derived from time-frequency decomposition of a time series during a small time window, corresponding to the burst event. Adults typically exhibit beta bursts with a stereotyped wavelet-like shape in the time domain (Sherman et al., 2016; Bonaiuto et al., 2021; Brady and Bardouille, 2022), which causes the observed features in the TF domain (Jones, 2016; Sherman et al., 2016; Szul et al., 2022). To determine the underlying cause of age-related changes in TF-based features, we compared the waveform shape of beta bursts between 9- and 12-month-olds with adults. The time window for waveform extraction was determined by the FWHM of lagged coherence averaged within the age-specific beta band, yielding 5 cycles (344 ms) for 9-month-olds, 5.2 cycles (341 ms) for 12-month-olds, and 5 cycles (233 ms) for adults. Bursts identified in each age group had a wavelet-like median waveform shape with a strong central negative deflection and surrounding positive deflections (Sherman et al., 2016; Bonaiuto et al., 2021) as well as more peripheral peaks (Brady and Bardouille, 2022; Szul et al., 2022), but there was great variability around this median (Fig. 6*a–c*). Infant bursts had a greater median waveform amplitude, and because they had a lower peak frequency, had slightly longer waveforms than adult bursts (Fig. 6*a–c*). However, after normalization and dynamic time warping (Fig. 6*d*,*e*) (Giorgino, 2009), bursts from both infant groups were revealed to have the same qualitative median waveform shape as adult bursts (Fig. 6*f*,*g*). The normalized distance between the infant burst waveforms and those of adult bursts was slightly lower in 9-month-olds than at 12 months (9m: 0.023, 12m: 0.033). Despite differences in TF-based burst features between age groups, beta bursts have a common median waveform shape across ages, suggesting that they are generated via similar mechanisms that change only quantitatively with age.

### The same burst motifs are increasingly rate-modulated during movement from infancy to adulthood

Although the warped burst waveforms were very similar across age groups, there was a considerable amount of variability around the mean waveform shape. It has been suggested that this variability reflects variability in function (Szul et al., 2022); therefore, we then examined how waveform variability changes with age and thus might drive developmental changes in motor control. We applied PCA to the warped, aligned waveforms from all age groups (364,728 bursts overall; 9m: 73,741; 12m: 94,856; adult: 196,131) to identify common motifs that explain burst waveform variance across ages. We extracted 20 PCs, of which 14 explained 80.42% of the waveform variance. To determine which components significantly contributed to waveform variability, we used a permutation test, resulting in 10 significant components (PCs 1-10; *p* < 0.001). Each of these dimensions defined changes in the amplitude or relative amplitude of the central negative deflection, surrounding positive deflections, and peripheral deflections.

**Figure 7.**
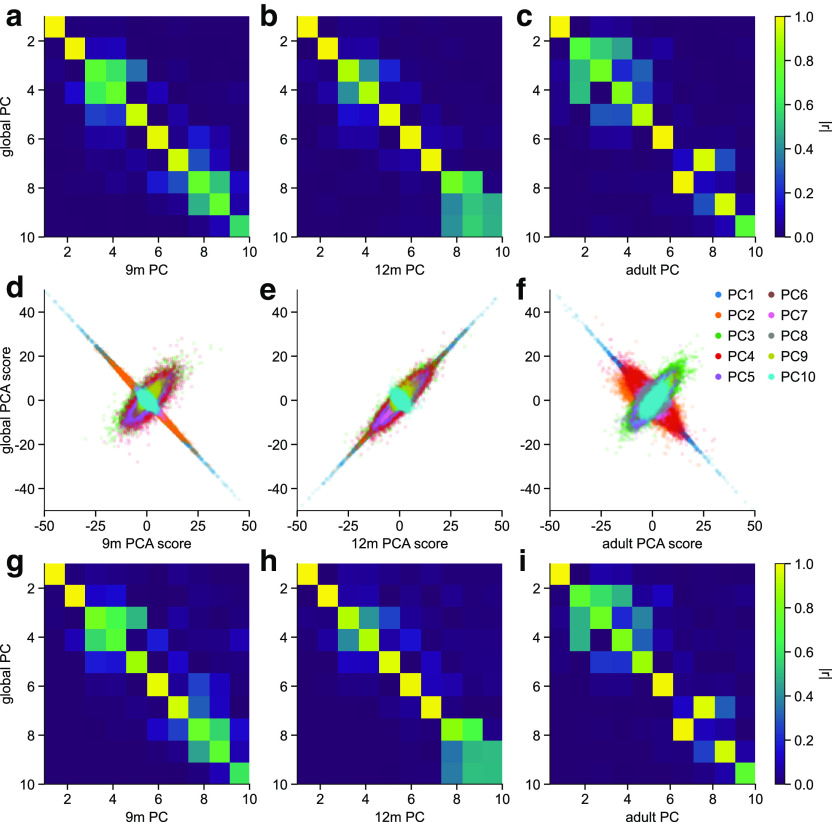
Global versus age group-specific PCA. ***a***, Absolute value of the correlation coefficient between eigenvectors from the PCA ran only on 9-month-old bursts, and those from the global PCA. ***b***, ***c***, Same as in ***a***, for 12-month-olds (***b***) and adults (***c***). ***d***, For each burst detected in 9-month-olds, the score for each PC from PCA ran on only the 9-month-old bursts (*x* axis) versus the score from the PCA ran on all bursts over all ages. ***e***, ***f***, Same as in ***d***, for 12-month-olds (***e***) and adult participants (***f***). ***g***, Absolute value of Pearson's correlation coefficient between scores for 9-month-old infant bursts from PCA applied only to 9-month-old bursts and the global PCA, for each PC. ***h***, ***i***, Same as in ***g***, for 12-month-olds (***h***) and adult participants (***i***).

We used a single PCA applied globally to all warped burst waveforms from all ages to compare waveforms between age groups using a common set of dimensions. However, it could be that each component describes waveform variance in a single age group. To verify that the identified burst waveform motifs were not biased toward an overrepresentation of waveform variability from one or two age groups, we ran separate PCAs on warped bursts from each age group and compared the resulting PCs and burst scores with those from the global PCA applied to all bursts from all age groups. PCs 1-8 were highly similar across ages, both in terms of eigenvectors (9m: *r* = 0.73-1.0; 12m: *r* = 0.79-1.0; adult *r* = 0.71-1.0; Fig. 7*a–c*), and burst scores (9m: *r* = 0.72-1.0; 12m *r* = 0.84-1.0; adult: *r* = 0.73-1.0; Fig. 7*d–i*). For PCs 1-6, the most similar component from each group PCA was the corresponding one of the global PCA, indicating that for these components, not only was the waveform motif very similar, but the relative percentage of variance explained was the same across ages. PCs 7 and 8, however, were reversed in order for the adult PCA, meaning that these motifs explained different amounts of variance in the infant versus adult waveforms. Two of the significant components, PCs 9 and 10, mainly represented waveform variability in the adult participants (PC 9: 9m eigenvector *r* = 0.75, burst score *r* = 0.74; 12m eigenvector *r* = 0.60, burst score *r* = 0.67; adult eigenvector *r* = 0.95, burst score *r* = 0.94; PC 10: 9m eigenvector *r* = 0.57, burst score *r* = 0.61; 12m eigenvector *r* = 0.48, burst score *r* = 0.51; adult eigenvector *r* = 0.72, burst score *r* = 0.74). All but two of the significant burst waveform motifs were therefore common among infants of 9 and 12 months, as well as adults.

**Figure 8.**
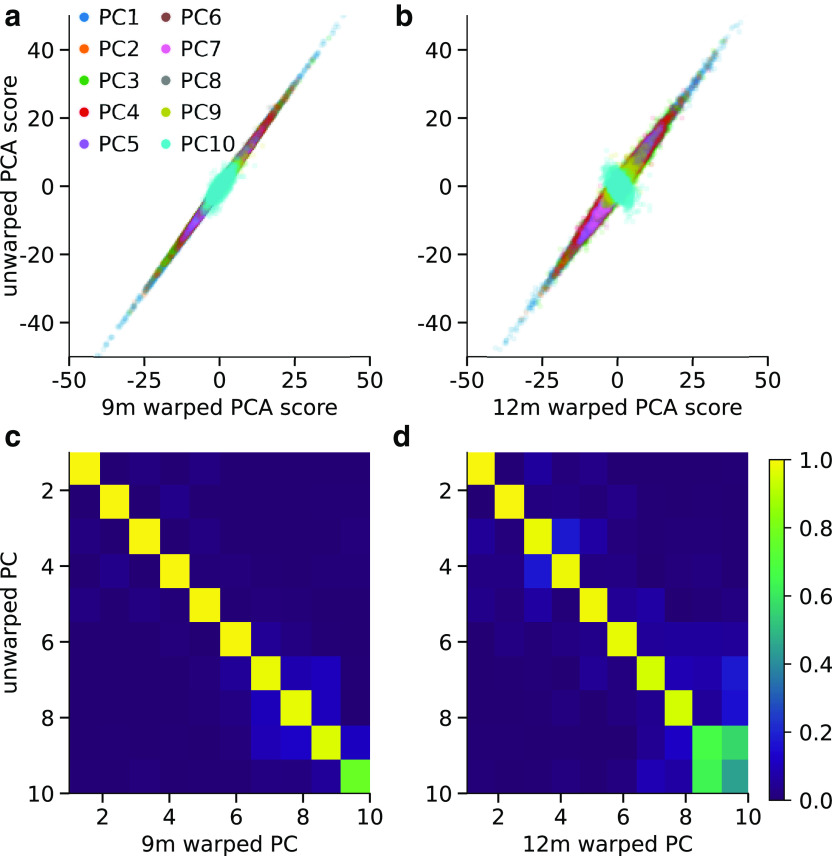
PCA on warped versus unwarped infant burst waveforms. ***a***, For each burst detected in 9-month-olds, the score for each PC from PCA ran on only the warped 9-month-old bursts (*x* axis) versus the score from the PCA ran on only the unwarped 9-month-old bursts. ***b***, Same as in ***a***, for 12-month-olds. ***c***, Absolute value of Pearson's correlation coefficient between scores for 9-month-old infant bursts from PCA applied only to warped 9-month-old bursts and the PCA applied to unwarped 9-month-old bursts, for each PC. ***d***, Same as in ***c***, for 12-month-olds.

To determine whether the dynamic time warping applied to infant burst waveforms had an effect on the PCA results, we compared burst scores for each PC with those obtained from running PCA on the unwarped infant bursts. This revealed that burst scores for PCs 1-8 were very highly correlated between the warped and unwarped waveforms (9m: *r* = 0.97-1.0; 12m: *r* = 0.95-1.0; Fig. 8). PCs 9 and 10 were less highly correlated between warped and unwarped waveforms (PC 9: 9m *r* = 0.96, 12m *r* = 0.77; PC 10: 9m *r* = 0.67, 12m *r* = 0.57), and they were also the ones identified as mainly representing variability in the adult burst waveforms. This demonstrates that dynamic time warping does not appreciably modify patterns of burst waveform variability. We therefore proceeded in the following analyses using PCs 1-8 from the global PCA run on warped burst waveforms from all age groups.

**Figure 9.**
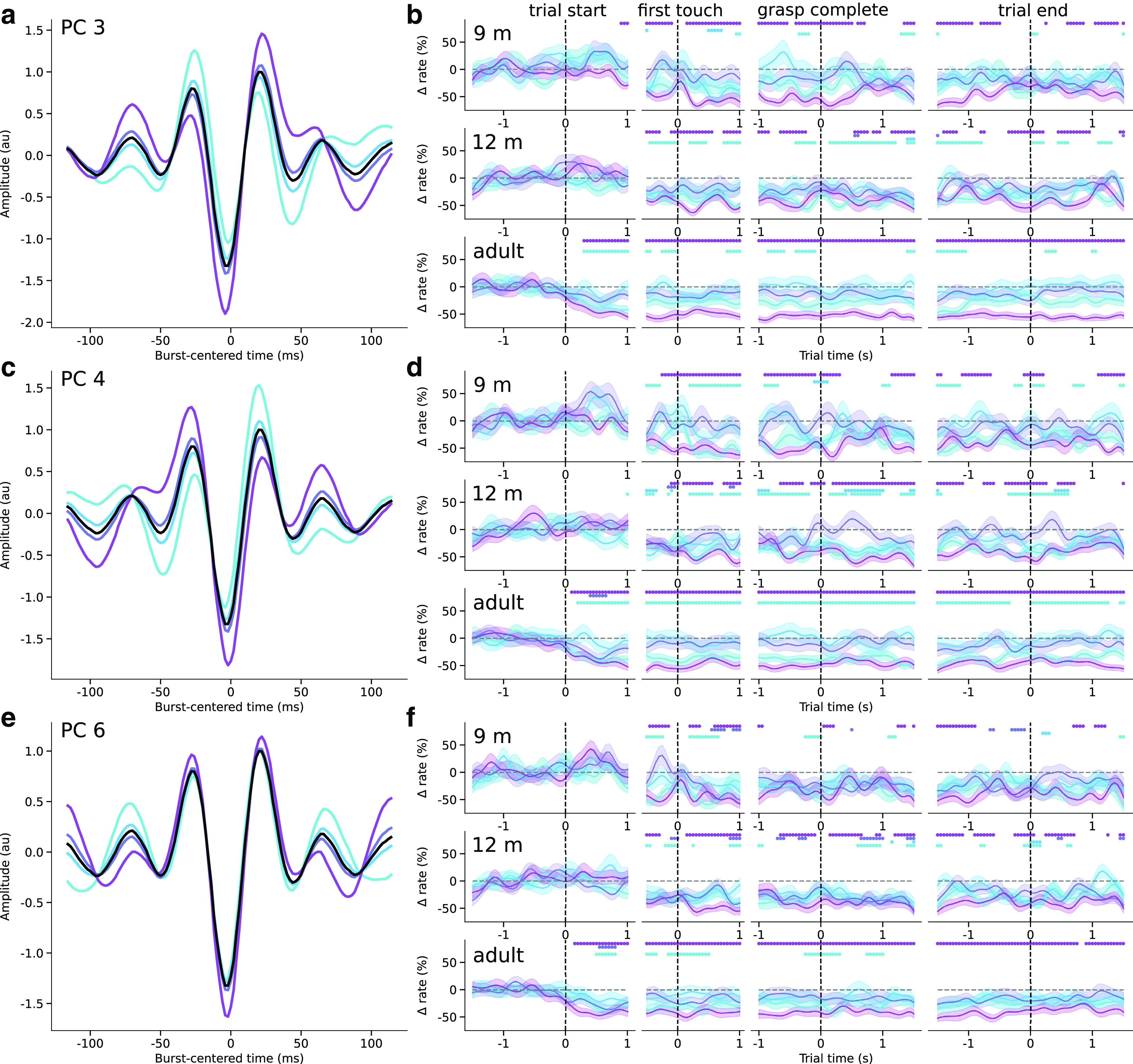
Contralateral burst waveform motifs are increasingly rate-modulated during movement from infancy to adulthood. ***a***, The mean normalized and warped waveforms of beta bursts with scores in four quartiles of PC 3 scores (colored lines) and the mean overall burst waveform (black). ***b***, The mean baseline-corrected rate of bursts with scores in each PC 3 score quartile (colored lines; shaded area represents SEM) over the course of the (columns, from left to right) trial start, first touch, grasp completion, and trial end epochs in the contralateral center cluster for 9-month-old (top row), 12-month-old (middle row), and adult (bottom row) participants. Colored dots represent where the burst rate in the corresponding score quartile is different from baseline. ***c***, ***d***, Same as in ***a***, ***b***, for PC 4. ***e***, ***f***, Same as in ***a***, ***b***, for PC 6.

**Figure 10.**
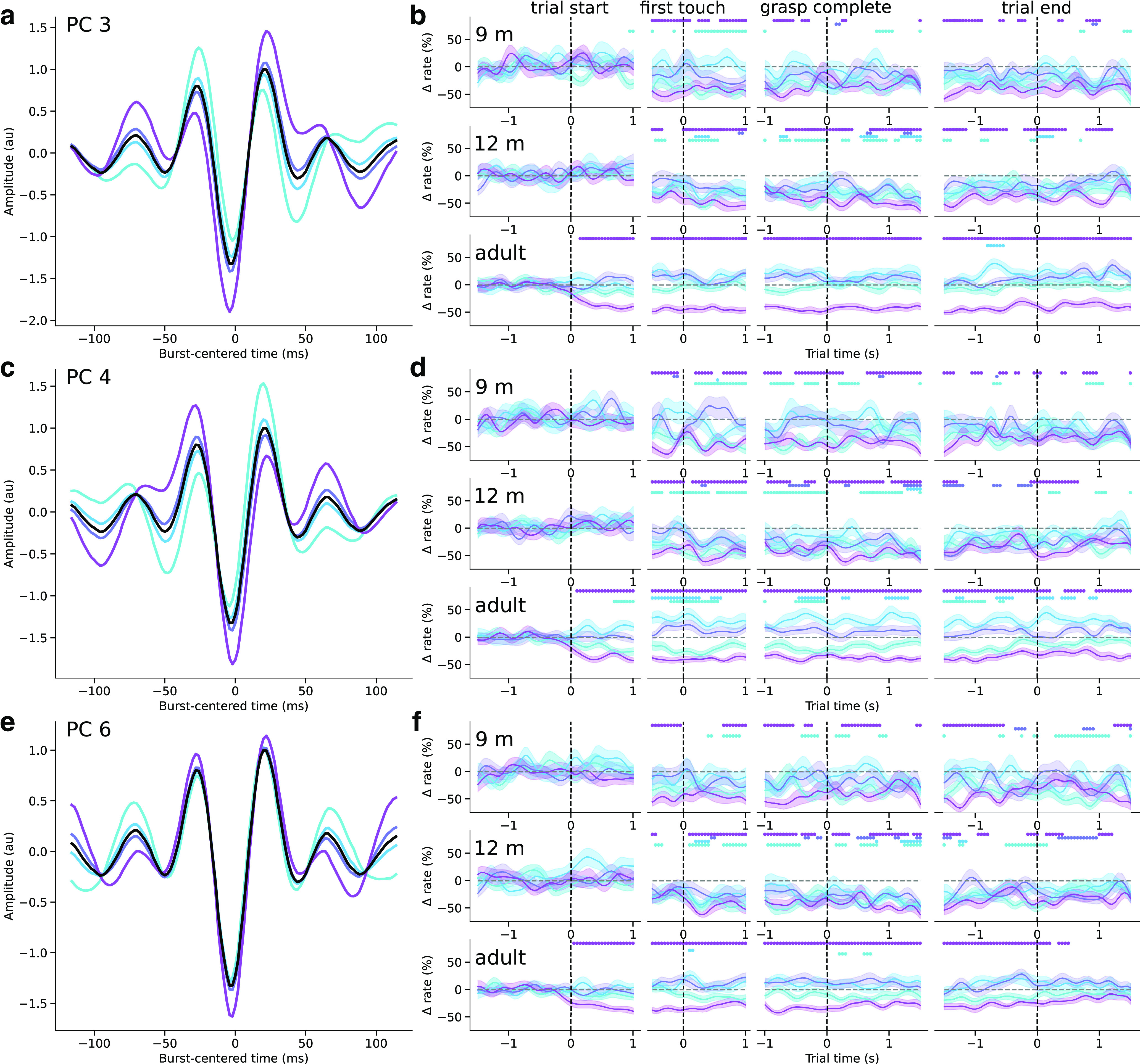
Ipsilateral beta burst motifs rate-modulated differently from contralateral motifs during movement in adulthood, but not infancy. ***a***, The mean normalized and warped waveforms of beta bursts in with scores in four quartiles of PC 3 scores (colored lines) and the mean overall burst waveform (black). ***b***, The mean baseline-corrected rate of bursts with scores in each PC 3 score quartile (colored lines; shaded area represents SEM) over the course of the (columns, from left to right) trial start, first touch, grasp completion, and trial end epochs in the ipsilateral central cluster for 9-month-old (top row), 12-month-old (middle row), and adult (bottom row) participants. Colored dots represent where the burst rate in the corresponding score quartile is different from baseline. ***c***, ***d***, Same as in ***a***, ***b***, for PC 4. ***e***, ***f***, Same as in ***a***, ***b***, for PC 6.

We then further analyzed three components based on significant changes in the mean burst score in the action execution epochs (no components were significantly modulated in the action observation condition), thus selecting dimensions along which the mean burst shape varied systematically over the course of the trial. Each of these components defined dimensions along which the waveform shape varied markedly from the median waveform (Figs. 9*a*,*c*,*e*, 10*a*,*c*,*e*). In each of these dimensions, the amplitude of peaks surrounding the central negative deflection, and that of the central deflection itself varied, but the most striking feature of each of the three components is that they represent waveforms with additional peripheral peaks and waveform asymmetry. PCs 3 and 4 defined motifs in which the asymmetry between the surrounding positive deflections and the magnitude of the central negative deflection varied (Fig. 9*a*,*c*), and PC 6 mainly represented changes in the amplitude of the deflections. The mean burst waveform score for each of these components decreased just before or after toy contact, and further decreased during the movement. Hence, not only did the overall burst rate decrease during the movement, but the mean burst waveform shape systematically changed throughout the task.

The systematic changes in overall burst rate and mean waveform shape do not indicate whether there was an increase in bursts with low component scores, or a decrease in bursts with high component scores. To investigate whether different waveform motifs were differentially rate-modulated during movement, we therefore examined the burst rate according to the waveform shape in the execution condition. For each of the three selected PCs, we binned bursts into four quartiles based on their score along that dimension, as well as the time during the trial in which they occurred. We then computed the burst rate for each PC score quartile, baseline-corrected the burst rates, and used permutation tests to determine significant deviations from the baseline. Along PCs 3, 4, and 6, bursts with scores in the first and fourth quartiles decreased in rate contralaterally during the movement compared with baseline, whereas those in the second and third quartiles (with waveform shapes closer to the median waveform) were not rate-modulated (Fig. 9*b*,*d*,*f*). Interestingly, the decrease in rate of bursts in the first and fourth quartiles occurred just after the onset of the trial in adults, and only just before or after the moment the hand touched the toy in 9- and 12-month-olds. Moreover, this decrease was significantly different from the baseline rate over longer periods of time in adults than in infants. Ipsilaterally, in adults, for the most part, only bursts in the fourth quartiles of PCs 3, 4, and 6 decreased in rate during movement (Fig. 10), and for PC 4, bursts closer to the median waveform actually increased in rate during movement (Fig. 10*d*). In infants, the rate of ipsilateral bursts in the first and fourth quartiles of each selected PC decreased during movement, similar to contralateral bursts. The other significant components were not reliably differentially rate-modulated during action execution in any age group, or only in adults. Beta bursts therefore occur within specific burst waveform motifs, some of which reduce in rate increasingly earlier during movement from infancy to adulthood, and others which are differentially rate-modulated ipsilateral to movement only in adulthood.

## Discussion

This study provides insights into the developmental trajectory of beta bursts and their potential role in sensorimotor processing. Findings demonstrate that infant beta activity, similar to adults, manifests as transient bursts with diverse spectral and temporal features, albeit at a lower peak frequency. Beta burst feature lateralization increases between 9 and 12 months, and further into adulthood. Infant beta bursts exhibit a mean waveform shape similar to adults and occur within a set of common motifs at all ages, indicating a possible shared mechanism of generation. Interestingly, several motifs classify beta bursts into those that decrease in rate during movement and those whose rate remains static or slightly increases, suggesting that different burst types are involved in distinct sensorimotor processes. Moreover, our results demonstrate that the bursts with waveforms furthest from the mean decrease more in rate and earlier during movement with age. In adults, the burst rate of certain motifs is differentially modulated ipsilateral and contralateral to the hand used for grasping, but infant beta bursts in these motifs are bilaterally rate-modulated during movement. Together, these findings highlight the complexity and diversity of beta bursts and their potential role in sensorimotor development.

The fact that infant and adult beta bursts have qualitatively similar mean waveform shapes suggests that they are generated by the same mechanism, which changes in a quantitative way with age. The dominant computational model of beta burst generation proposes that beta bursts are driven by temporally aligned synaptic inputs to deep and superficial layers that drive intracellular current in opposite directions within a cortical column (Sherman et al., 2016; Law et al., 2022). This model was based on the somatosensory cortex, but experimental support for the model has been found in the human motor cortex (Bonaiuto et al., 2021; Szul et al., 2022). The primary motor cortex receives layer-specific projections from different thalamic motor nuclei that relay information from the basal ganglia and cerebellum (Kuramoto et al., 2009, 2015; Hooks et al., 2013), as well as from thalamic sensory nuclei (Ohno et al., 2012; Hooks et al., 2013; Kuramoto et al., 2009, 2015). These projections may account for the stereotypical mean burst waveform shape, but the motor cortex also receives lamina-specific inputs from the sensory and frontal cortices (Rouiller et al., 1993; Mao et al., 2011; Hira et al., 2013; Hooks et al., 2013), which may account for the observed burst waveform variability (Szul et al., 2022). Myelination of inter-regional fiber tracts is particularly intensive during the first year of life (Dubois et al., 2014), which shapes neurophysiological activity (Adibpour et al., 2017); therefore, maturation of thalamocortical and corticocortical projections may explain differences in the peak frequency of infant and adult beta bursts.

Our results suggest that different types of beta bursts are involved in different sensorimotor mechanisms. Interestingly, we observed that bursts with certain waveforms decreased in rate earlier during movement in adults compared with infants. This may be because of the increasing use of visual information for movement preparation and planning and, more generally, a shift from reflexive to prospective control of movement (von Hofsten, 1993; van der Meer et al., 1994; Witherington, 2005). However, in this study, adults performed faster reaching movements than infants; therefore, the earlier burst rate decrease we observed in adults may simply reflect the fact that their hand was closer to the toy at the same time post-trial start.

Consistent with our behavioral results, young infants exhibit an ambidextrous pattern of hand usage (Corbetta and Thelen, 1999; Rönnqvist and Domellöf, 2006), but a bias for using one hand emerges between 12 and 18 months (Fagard and Marks, 2000; Hinojosa et al., 2003). While we observed a change in burst feature lateralization between 9 and 12 months, the overall burst rate was only lateralized in adults. Sensorimotor activity is not hemispherically lateralized in neonates (Erberich et al., 2006), which may be why there were no hemispheric differences in burst features or rate at 9 months. The increasing lateralization of beta burst activity between 9 months and adulthood may be because of the decrease in interhemispheric functional connectivity between motor cortices that occurs during the first year of life (Xiao et al., 2018), and the ongoing development of interhemispheric inhibition in childhood (Ciechanski et al., 2017). However, the pattern of activity that we observed in adults has implications for the functional role of beta activity. If beta activity is purely inhibitory in nature (Zhang et al., 2008; Picazio et al., 2014), one would expect a decrease in contralateral beta activity during movement, thus disinhibiting the limb to be moved, and either no change or an increase in ipsilateral beta activity. Consistent with this view, we observed a decrease in overall burst rate contralaterally in adults, and no change in overall ipsilateral burst rate. However, we found four main types of rate lateralization in different burst motifs: bursts with a static rate bilaterally, bursts that remained static contralaterally and increased in rate ipsilaterally, bursts that decreased in rate contralaterally and were static ipsilaterally, and bursts that decreased in rate bilaterally. These findings suggest that the role of beta activity in sensorimotor processes is more complex than a simple inhibitory effect.

Burst rate and beta power were only modulated during action observation in adults. Previous work has found a reduction in α/μ activity during action observation in infants and adults (Marshall and Meltzoff, 2011; Marshall et al., 2011; Cannon et al., 2014; Filippi et al., 2016), but beta activity has only been shown to be modulated during action observation in older infants (van Elk et al., 2008) and adults (Press et al., 2011). Motor simulation theory suggests that observation of someone else performing an action activates an internal sensorimotor simulation of performing that same action (Jeannerod, 2001; Miall, 2003; Wolpert et al., 2003; Press et al., 2011). If certain burst types are related to the activation of internal models, one possible explanation for this finding is that these internal models are not yet well developed in infants and are therefore not activated during action observation. This is in line with the increasing amount and earlier timing of rate modulation of these burst motifs from infancy to adulthood during action execution.

In accordance with the few previous studies that have identified age-specific beta frequency bands in infancy (Meyer et al., 2016; Rayson et al., 2022), we found the peak beta frequency to be ∼15 Hz in 9- and 12-month-old infants. One factor that may have contributed to the lack of previous research on infant beta activity is the fact that, in infants, muscle artifacts from jaw and arm movements appear at 15 Hz in peripheral electrodes, spectrally overlapping the sensorimotor beta frequency range (Georgieva et al., 2020). However, it is unlikely that our results are driven by these artifacts because the spatial topography of lagged coherence in the identified beta band localizes very strongly to the central electrodes, and we found a decrease in burst rate during movement. Beta peak frequency increases with age in older children and adults (Trevarrow et al., 2019; Johnson et al., 2020), suggesting that this spectral shift is an ongoing developmental process, similar to that observed in α/μ (Berchicci et al., 2011).

The present study has several limitations worth noting. Our results suggest developmental shifts in the strength, timing, and duration of different synaptic drives to the sensorimotor cortex, thus modulating the features and rate of sensorimotor beta bursts. However, without any measure of anatomic or functional connectivity, it is impossible to know where these projections originate from. In this study, the actual 3D trajectories of the arm and hand during performed and observed movements were not quantified, but the movements were quite complex, limiting the granularity of the conclusions that can be drawn about the relationship between beta activity and sensorimotor processes. Very few adult subjects performed a small number of grasps with their nonpreferred (left) hand, whereas infants tended to use either hand, and therefore these results cannot shed light on lateralized control of the preferred versus nonpreferred hand. We analyzed EEG data, which is limited in spatial precision because of the effects of volume conduction and uncertainty regarding the exact position of each electrode with respect to the brain. Although this study included multiple age groups, it was cross-sectional. The ideal future study for determining the developmental role of beta bursts would therefore be longitudinal from infancy to early childhood, and involve MRI, MEG/EEG analysis in source space, and markerless kinematic tracking during simple movements with each hand in all age groups to determine the precise relationship between afferent brain regions driving beta bursts, burst characteristics, and movement. Finally, the burst detection approach that we use extracts waveforms centered on peaks of beta power, and therefore highlights high SNR temporal features around these peaks (Szul et al., 2022) at the expense of the small number of potentially longer-lived oscillatory bursts. Future approaches for burst analysis could use some form of phase information or measure of instantaneous frequency to tease apart these potentially two types of beta activity patterns.

Critically, several developmental disorders, such as attention deficit/hyperactivity disorder (in a subset of children) (Clarke et al., 2007, 2013) and autism spectrum disorder (Coben et al., 2008; Tierney et al., 2012), are associated with aberrant beta activity. Early diagnosis is crucial for identifying individuals who would benefit from therapeutic intervention (Landa, 2008; Rommel et al., 2015), with the most effective interventions implemented during sensitive periods of brain development. However, such interventions require sensitive biomarkers to determine their efficacy in their early stages because their behavioral effects may be delayed (Cioni et al., 2016; Finlay-Jones et al., 2019; Morris et al., 2023), and the notion of “aberrant beta activity” is undermined by the diversity of burst activity we observe. Burst waveform shape could therefore provide the required sensitivity by allowing detection of abnormal burst waveforms, altered rate-modulation of certain types of bursts, or atypical lateralization of burst features or rate, all of which would be masked by nonspecific measures of highly averaged beta power. More generally, comparison of beta burst activity in typical versus atypical motor development trajectories may be instrumental in teasing apart the different mechanistic functional roles of different burst types.
